# Characterisation and Application of Bio-Inspired Hybrid Composite Sensors for Detecting Barely Visible Damage under Out-of-Plane Loadings

**DOI:** 10.3390/s24165170

**Published:** 2024-08-10

**Authors:** Ali Tabatabaeian, Reza Mohammadi, Philip Harrison, Mohammad Fotouhi

**Affiliations:** 1 James Watt School of Engineering, University of Glasgow, Glasgow G12 8QQ, UK; 2 Faculty of Civil Engineering and Geosciences, Delft University of Technology, 2628 CN Delft, The Netherlands

**Keywords:** barely visible impact damage, hybrid composite sensor, visual inspection, bio-inspired mechanochromic composites

## Abstract

Traditional inspection methods often fall short in detecting defects or damage in fibre-reinforced polymer (FRP) composite structures, which can compromise their performance and safety over time. A prime example is barely visible impact damage (BVID) caused by out-of-plane loadings such as indentation and low-velocity impact that can considerably reduce the residual strength. Therefore, developing advanced visual inspection techniques is essential for early detection of defects, enabling proactive maintenance and extending the lifespan of composite structures. This study explores the viability of using novel bio-inspired hybrid composite sensors for detecting BVID in laminated FRP composite structures. Drawing inspiration from the colour-changing mechanisms found in nature, hybrid composite sensors composed of thin-ply glass and carbon layers are designed and attached to the surface of laminated FRP composites exposed to transverse loading. A comprehensive experimental characterisation, including quasi-static indentation and low-velocity impact tests alongside non-destructive evaluations such as ultrasonic C-scan and visual inspection, is conducted to assess the sensors’ efficacy in detecting BVID. Moreover, a comparison between the two transverse loading types, static indentation and low-velocity impact, is presented. The results suggest that integrating sensors into composite structures has a minimal effect on mechanical properties such as structural stiffness and energy absorption, while substantially improving damage visibility. Additionally, the influence of fibre orientation of the sensing layer on sensor performance is evaluated, and correlations between internal and surface damage are demonstrated.

## 1. Introduction

Low-velocity impact can happen during the manufacturing and in-service life of a composite structure, causing serious internal damage without a noticeable sign on the structure’s surface, often referred to as barely visible impact damage (BVID). This can pose different risks to structural integrity [1,2,3]. In recent years, structural health monitoring (SHM) has evolved into a multidisciplinary research field aiming to enhance the lifetime and maintenance of engineering systems. Various strain sensing technologies have been developed, including radio frequency identification (RFID) [4,5], fibre Bragg grating (FBG) sensors [1], electrical resistance measurements [6], and multifunctional polymer composite systems [7]. Additionally, novel machine learning methods for damage detection, such as sparse Bayesian learning (SBL) [8] and convolutional neural networks (CNNs) [9], have been introduced.

Inspired by the colour-changing abilities of biological skins, the exploration of novel mechanochromic materials has become one of the popular and important research directions, enhancing the competitive advantages over other smart devices. These bio-inspired mechanochromic materials could generate optical variations (transparency, fluorescence, and colour) in response to external stimuli, such as transverse loads, providing a direct and eye-detectable visual presentation of environmental variation to the users [10]. Bio-inspired mechanochromic sensors can be categorised into two main groups: (1) chemical-based and (2) physical-based sensors. In chemical-based sensors, the colour-changing process stems from the selective absorption and reflection of specific wavelengths of electromagnetic radiation [11]. On the other hand, physical-based sensors are connected to both the shape and refractive index of the material and not to its chemical properties [12]. An example of a physical sensor is a thin-ply hybrid composite that changes colour based on a bio-inspired design. This composite uses layers of fibres with varying strain-to-failure ratios, such as a layer of carbon fibres combined with a layer of translucent glass fibres. The changes in light absorption at the interfacial glass/carbon damaged area can generate a clear visual cue by which damage can be detected as an early warning to avoid catastrophic structural failure due to hidden damage. Czel and Wisnom [13] first introduced hybrid thin-ply composite sensors while studying pseudo-ductile behaviour in thin interlayer glass/carbon–epoxy hybrid composites. They noted a pattern during the specimens’ gradual failure. The translucent properties of the glass–epoxy layers made it possible to detect delamination visually. This observation led to the realisation that such composites could be used to sense surface damage on structures. Hybrid composite sensors were later applied for monitoring damage in carbon fibre-reinforced polymer (CFRP) composite panels under tensile [14], fatigue [15], and impact loadings [16]. Rev et al. [14] successfully demonstrated mechanical characterisation and the integration of such sensors to a real-life application demonstrator: a CFRP bicycle handlebar under a three-point bending test. Despite some prior research in this area, the sensing capabilities of hybrid composite sensors under out-of-plane loadings, such as static indentation and low-velocity impact, remain inadequately explored in the literature. This study aims to address this gap by presenting novel findings that contribute to understanding and enhancing the performance of these sensors in such loading conditions.

This paper demonstrates novel hybrid thin-ply glass/carbon sensors that improve the detection of low-energy impact damage in laminated composite structures. Following the design and manufacture of the sensor-integrated CFRP panels in Section 2 and Section 3, a series of quasi-static indentation and low-velocity impact tests are conducted on the composite specimens with and without the attached hybrid sensors, with three impact energy levels, namely, 12J, 18J, and 27J, and a detailed assessment of the damage evolution is carried out through non-destructive tests (NDTs), including ultrasonic C-scan and visual inspection (Section 4). The experimental investigation is divided into two parts: The first part focuses on the quasi-static indentation behaviour, providing reference data for designing low-velocity impact experiments. The second part investigates the sensor performance in enhancing the visibility of low-energy impact damage. Section 5 provides a detailed analysis of the results, covering the impact of sensor integration on mechanical properties like structural stiffness and energy absorption. It also examines the damage sensing capabilities of the sensor under static indentation and low-velocity impact loads, as well as the correlation between the sensor-activated area and the severity of the damage. Additionally, it explores how design parameters, such as the fibre orientation in the sensing layer, affect performance. Finally, a comparison between the static indentation and low-velocity impact tests is presented, and the key findings are summarised in Section 6.

## 2. Design Principles of the Sensor

**Figure 1:**
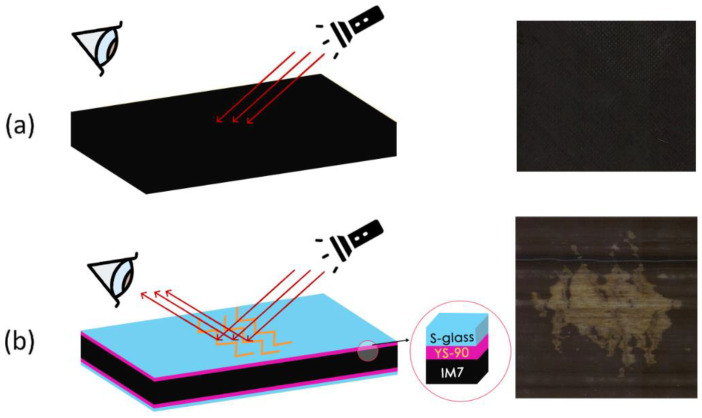
Schematic of (**a**) a reference specimen and (**b**) a sensor-integrated specimen, showcasing the sensor’s working principle and configuration (**left**). The images on the right show the post-low-velocity impact surface of the specimens, highlighting the mechanochromic function in the sensor-integrated sample.

Wisnom and colleagues [17] reported that well-designed thin-ply hybrids can develop multiple fractures of the higher modulus/lower strain constituent. This characteristic enables them to evade catastrophic failure and unstable delamination, thanks to the low-energy release rate primarily influenced by the thickness of the stiffer component layer within the hybrid structure. For example, in thin-ply glass/ultra-high modulus carbon hybrids, the cracks and delamination in the carbon ply are visible to the naked eye due to the translucency of the glass/epoxy plies. This study’s idea for designing hybrid sensors is based on a unique characteristic of the thin interlayer glass/carbon hybrid composites: a visible colour change occurs when subjected to strains surpassing a predefined threshold. As the sensor is applied onto the surface of the structure, it experiences similar strains as the underlying material. It comprises a carbon sensing layer and an outermost glass layer. As shown in Figure 1, a *reference* specimen (without a sensor) absorbs the light after being subjected to an impact load, indicating a dark appearance. In the *sensor* specimen, on the other hand, after the impact, when the strain goes beyond the failure strain of the carbon sensing ply, the carbon ply develops multiple fractures, reflecting the light from the damaged glass/carbon interface around the fractures in the carbon layer, making the damage visible.

Hybrid composite sensors can be designed considering different parameters that control the damage mechanisms, i.e., fibre fragmentation and/or delamination. These include the sensor material strain to failure, thickness, stacking sequence, and critical energy for delamination initiation. For example, different values of the sensor-to-substrate stiffness ratio can be achieved by either changing the thickness of the layers or using different composite prepreg materials. Another critical design parameter is the failure strain of the carbon sensing layer that controls the sensor activation time and strain threshold. The design of hybrid sensors for monitoring impact damage, i.e., selection of the thickness, materials, and lay-up, can be carried out by calculating the critical load levels for the three failure mechanisms of mid-plane delamination, impacted face compressive fibre failure, and back-face tensile fibre failure in a laminate under low-velocity impact. The equations associated with these failure mechanisms can be used simultaneously to evaluate the competition between primary damage modes and to design the sensor according to the desired level of damage detection [16].

In a laminated composite structure composed of different orthotropic layers under flexural loading through the thickness, plies with different fibre orientation tend to deform differently due to the bending stiffness and bending–twisting coupling effect. Normal (i.e., normal to the plane of ply) and shear stresses are developed at the interface between plies with different orientations. As the flexural deformation increases, these interlaminar stresses increase and exceed the critical values, causing delamination initiation. Despite shear and tensile matrix cracking, delamination leads to a high level of energy release rate, which can cause a sudden load drop in the load-displacement graph. Note that in this paper, the term “load drop” signifies a sudden change in the shape of a graph. The critical load for initiation of midplane delamination (F_d_) can be calculated using Equation (1) [18,19]:

(1) $${F_{d}}^{2}=\frac{8\pi^{2}Et^{3}G_{IIC}}{9(1-\vartheta^{2})}$$

where E, t, ϑ, and G_IIC_ are the effective homogenised Young’s modulus of the laminate in bending, thickness, Poisson’s ratio, and critical strain energy in mode II delamination, respectively. Therefore, the load associated with delamination initiation depends not only on the thickness ratio (t^1.5^) but also on the critical energy release rate (G_IIC_^0.5^) and Young’s modulus (E^0.5^) of the laminate.

Generally, fibre failure occurs much later in the fracture process than matrix cracking and delamination, which is considered a sign of plate perforation. There are two types of fibre failure, which are the result of compressive and tensile loads. Compressive fibre failure occurs on the impacted side, where contact and compressive bending stresses dominate. On the impacted side, both normal stress and transverse shear stress may be responsible for the fibre failure. On the other hand, tensile fibre failure occurs on the back face of the laminate (opposite side of the impact), where the tensile bending stresses are high. Therefore, to predict critical load associated with the fibre failure, two approaches should be implemented based on classical plate theory for tensile fibre failure and integration of contact and classical plate theory for compressive fibre failure [16,20]. The detailed analytical formulation is not provided here, as it is not the central focus of this paper.

Delamination is identified as the most critical damage mode in composites exposed to transverse impact. Since this study focuses on low-energy impact energies that cause BVID, delamination is of significant interest here. Moreover, the results of research by Hallet et al. [21,22] on quasi-isotropic composite laminates with similar materials, stacking sequence, and geometries to those of this study showed that under static indentation and low-velocity impact, delamination always occurs before both back-face tensile fibre failure and impacted side compressive fibre failure. In this case, delamination will be the baseline for designing hybrid composite sensors for this study. Therefore, the idea is to prevent mid-plane delamination damage as the first damage mode and instead trigger fibre failure in the low-strain sensor material (carbon sensing layer) as the first active mode.

**Figure 2:**
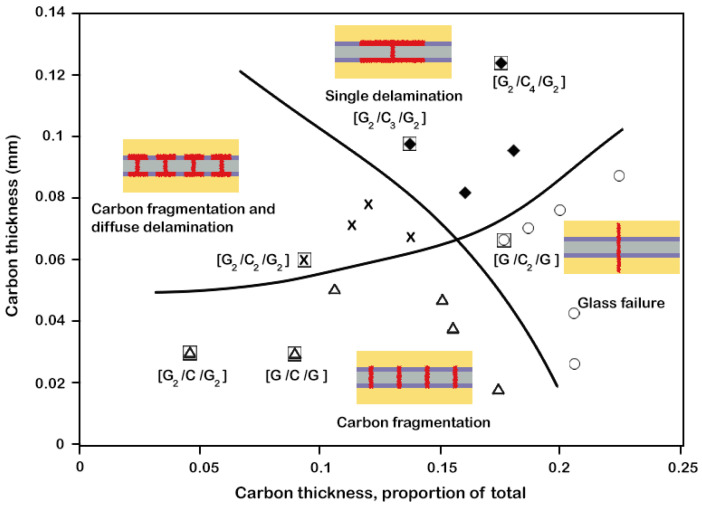
Categorisation of different damage modes as a function of absolute and relative thickness of carbon layers (reprinted with permission from Ref [23]).

The sensor can be integrated by either co-curing or retrofitting by bonding onto finished parts. Depending on the curing temperature of the sensor and substrate prepreg materials, the co-curing itself can also be done through either a one-step or a two-step curing procedure. By co-curing, the sensor acts as a structural sensing layer, while by retrofitting, it acts as a discrete sensor on the structure. It should be noted that the thickness of the sensing material must be thin enough to exhibit failure with fragmentation and dispersed delamination. This was studied in detail in work by Jalalvand et al. [23], where a damage mode map was developed using numerical damage analysis of hybrid glass/carbon composites with different thicknesses of the carbon layer (see Figure 2).

## 3. Materials and Manufacturing

Two main groups of composite specimens were manufactured, including reference (*reference*) and sensor-integrated (*sensor*) laminates. The *reference* laminate had a quasi-isotropic stacking sequence, [45/0/90/−45]_4s_, made of a unidirectional IM7 carbon/8552 epoxy. The direction of unidirectional fibre orientation that runs parallel to the longer side of the plate is considered as 0°. Each sensor consisted of a single layer of YS-90A carbon prepregs placed between the core laminate and a single layer of S-glass/913 epoxy prepreg with a 90° orientation. Moreover, to investigate the influence of the fibre orientation of the sensing layer, different fibre directions were used in carbon sensing ply of *sensor* samples. According to the ASTM D7136 standard test method [24], all samples were manufactured with dimensions of 100 mm × 150 mm. All composite specimens were fabricated using the conventional process of prepreg composite manufacturing. Following hand lay-up, a standard bagging method was applied on a flat aluminium tool plate. Additional silicone sheets were placed on top of the laminates to ensure a smooth top surface and an even pressure distribution in the autoclave. For *sensor* samples, due to different curing temperatures, the substrate was manufactured first, followed by applying the sensor onto the substrate (both sides) and co-curing the entire structure under the curing temperature required for the sensor. This manufacturing method allowed for investigating the influence of different attachment methods on the properties and potential applications of the laminates. Specifically, the separate application of the sensor (the two-step curing process) could potentially broaden the range of applications for which these laminates could be used.

**Figure 3:**
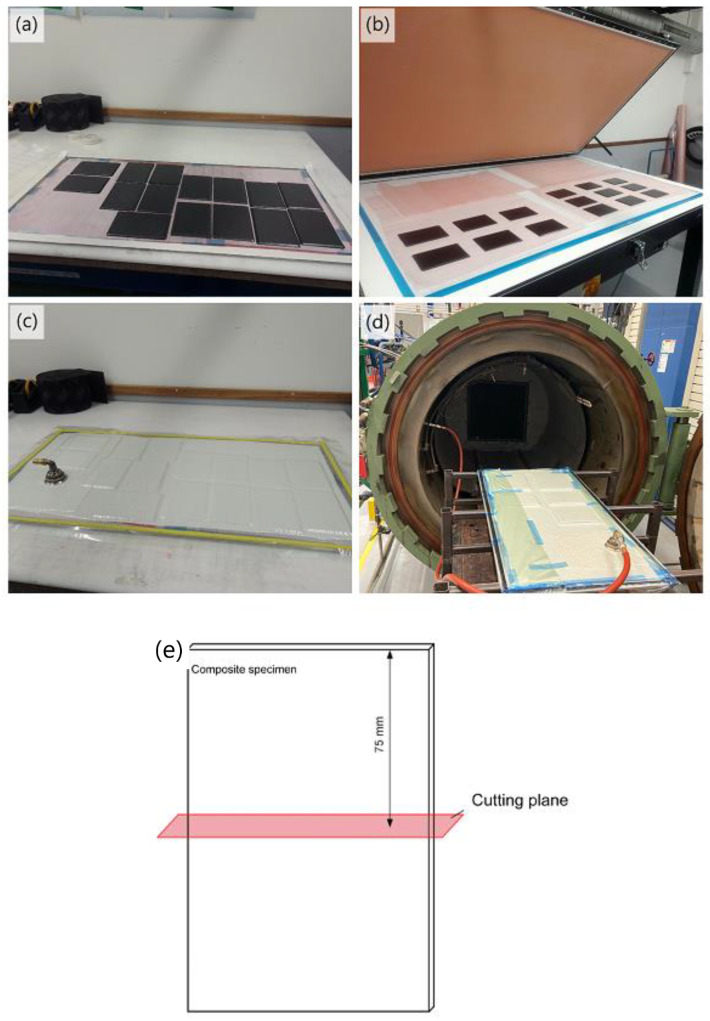
Manufacturing process: (**a**) cutting the prepregs into standard specimen size and stacking the layers, (**b**) applying pressure after stacking every four layers to ensure there is no air or bubbles, (**c**) completing the vacuum-bag lay-up, (**d**) curing at autoclave under desired temperature and pressure, (**e**) cutting plane for examining the cross-section of the impacted specimen.

The substrate was initially cured at 110 °C for 60 min, followed by 180 °C for 120 min under a constant 0.7 MPa pressure. Then, on the next day, a second curing procedure for 60 min at 125 °C, with 0.7 MPa applied pressure, was conducted. These were used according to the recommended curing cycles for IM7/8552, IM7/913, and S-glass/913 prepregs [19,25] (see Table 1). Figure 3 shows the manufacturing process followed in this study. After conducting low-velocity impact tests, some impacted samples were cut to examine the internal damage at the cross-section under the microscope.

## 4. Test Methods

### 4.1. Quasi-Static Indentation

Quasi-static indentation testing can be used as an alternative for low-velocity impact testing for FRP composites; however, several factors affect how comparable the results are, for instance, the impact energy and stiffness of the laminate [27,28,29]. This section uses indentation testing to estimate the impact properties and the sensing layer activation energy. The information from the indentation testing was then used to determine suitable impact energy values for the drop weight impact testing. As the purpose of the sensing layer is to increase the visibility of BVID, the main objective for performing indentation tests is to determine the energy range at which BVID will occur.

**Figure 4:**
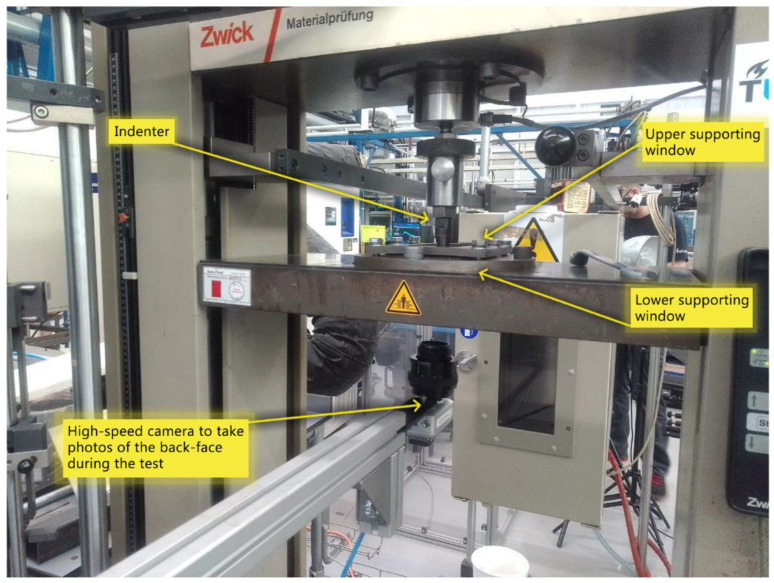
Indentation test setup.

The out-of-plane indentations were applied vertically on the front surfaces of the specimens at a constant indentation rate of 2 mm/min using a steel hemispherical indenter with a diameter of 16 mm, in accordance with the ASTM D7136-07 standard for drop weight impact damage resistance on FRP composites, ensuring the indentation test results match drop weight impact testing as closely as possible. The dynamic and rate effects of the laminate were considered to be minimal at this loading rate [21]. The specimen was positioned centrally over a 75 mm × 125 mm supporting window and was clamped in position, ensuring that the clamping base and the specimen were central to the indenter. Experiments were continued until a significant drop in force was observed, and the force did not continue to rise after the drop. This is the termination load drop, marked by an audible cracking sound. All tests (indentation and impact) were performed at room temperature. As shown in Figure 4, and a camera was placed beneath the supporting window to capture the initiation and propagation of the damage as well as sensor activation. The camera was set to take a photo every one second, collecting an image-based dataset during the whole test time.

### 4.2. Low-Velocity Impact

**Figure 5:**
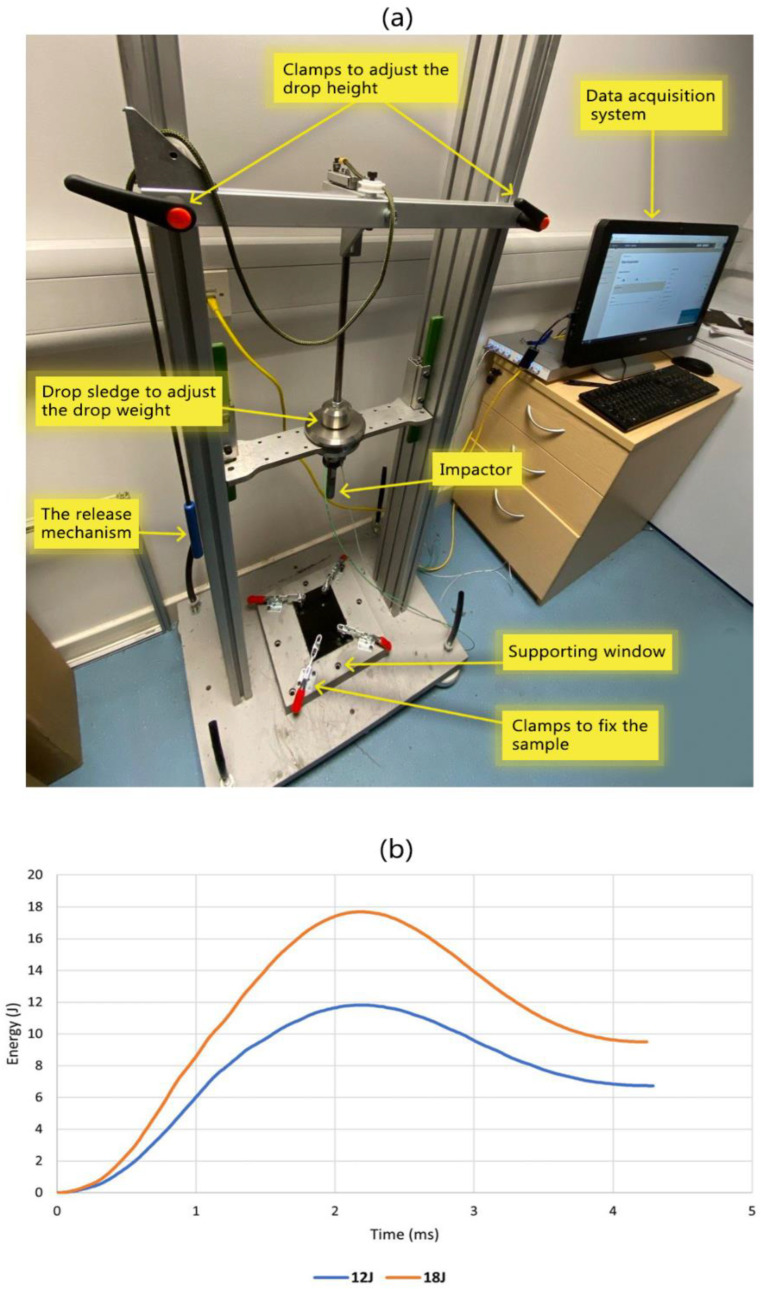
Impact test setup: (**a**) different parts of the custom-designed drop tower apparatus; (**b**) calculated impact energy at 12J and 18J impact tests from force–time results. Peak values are seen to match the corresponding test energy, demonstrating an effective setup.

Based on the indentation results, three impact energy levels were considered (12J, 18J, and 27J) and carried out according to the ASTM D7136 standard [21]. The drop mass was chosen to ensure the lowest drop energy of 12J would not require a drop height of less than 300 mm, ensuring test standards were met. As a result, the drop mass was recorded to be 3.94 kg, including all components on the dropping sledge (see Figure 5a). A Kistler 9333 load cell, which is capable of recording impact forces of up to 50 KN, was used. The maximum data sampling rate for the load cell is 62,500 samples per second. This rate was chosen as impact durations were expected to be less than 10 ms; therefore, it was desirable for sampling to be as high as possible to ensure good resolution in the results. A lowpass filter embedded within the data acquisition software was used to ensure the results had minimal noise. This was a Butterworth filter, and the cut-off frequency was set to 3000 Hz. This frequency was chosen by performing multiple drop tests with different filter frequencies. The frequency was chosen so that the section of the curve indicating elastic rebound was smooth with minimal noise, yet the oscillatory areas resulting from specimen damage remained visible.

To determine the drop height for the desired impact energy, $E_{impactor}$, the gravitational potential energy equation was used (Equation (2)), where m is the mass of the sledge, g is the gravitational acceleration, and h is the drop height measured from the specimen’s top surface to the impactor’s point of first contact. The energy lost due to friction was assumed to be negligible. The acceleration and velocity can then be calculated using Equations (3) and (4). Displacement was calculated using Newton’s second law and the integral of the velocity (Equation(5)):

(2) $$E_{impactor}=mgh$$

(3) $$a\left(t\right)=g-\frac{F\left(t\right)}{m}$$

(4) $$v\left(t\right)=v_{1}+\int_{0}^{t}a\left(t\right)dt$$

(5) $$x\left(t\right)=\int_{0}^{t}v\left(t\right)dt$$

Due to potential variability in dynamic loading tests, all impact tests were conducted twice to ensure the reliability of the results. Post-impact specimens were labelled, and images were taken of both sides to enable visual inspection of the impacted and non-impacted surfaces. Plots of the impact energy vs. time were made to confirm a correct drop tower setup, and that post-processing had been carried out effectively, as shown in Figure 5b. The peak values on this plot represent the point when all the impact energy has been transferred into the test specimen. As seen below, the peak energy values closely match the impact energy the impact test was set at. This confirms an effective setup and post-processing of the force–time results.

### 4.3. Visual Inspection, Ultrasonic C-Scan, and Image Processing

In the present research, two NDT methods were used to further analyse the damage after completing low-velocity impact tests. First, the impacted and non-impacted faces of all samples were scanned using a Canon C257i scanner (visual inspection). After that, all samples were C-scanned using a DolphiCam2 camera [30]. A coupling agent (gel) was used to enhance the transmission of ultrasound waves between the transducer and the specimen’s surface, facilitating accurate imaging and defect detection. Finally, the damage area was measured and recorded from the image obtained.

**Figure 6:**
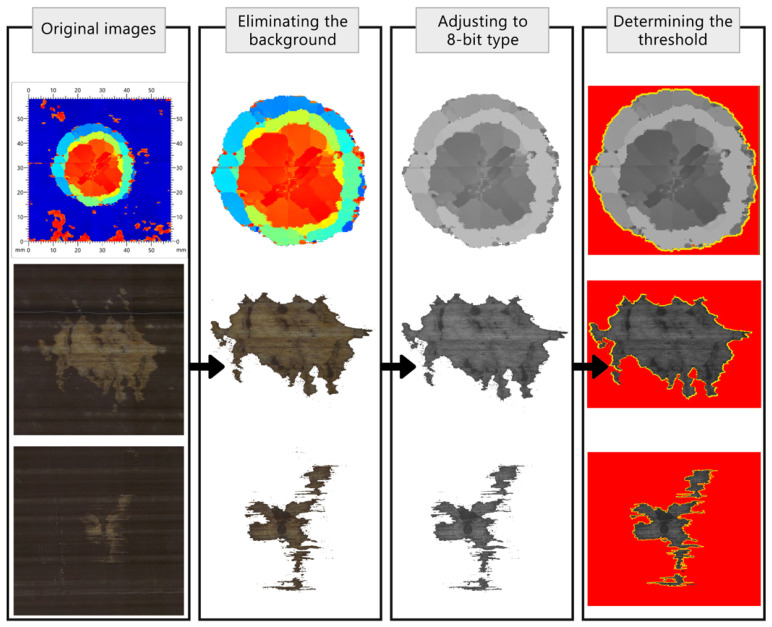
The image processing method to measure the C-scanned area and sensor-activated areas.

Following the collection of image-based data from the front face, back face, and C-scan of the samples impacted at various energies, image processing was performed using ImageJ software to measure the sensor-activated area on the surface as well as the C-scanned area. This analysis aimed to establish a correlation between surface and internal damage. The image processing procedure involved utilising Adobe Photoshop software (version 25.6)to eliminate the background, followed by adjusting the image type to 8-bit using ImageJ software. Subsequently, the threshold was determined, and the area was measured using the Region of Interest manager toolbox. This process is illustrated in Figure 6.

## 5. Results and Discussion

### 5.1. Quasi-Static Indentation

#### 5.1.1. Global Behaviour

**Figure 7:**
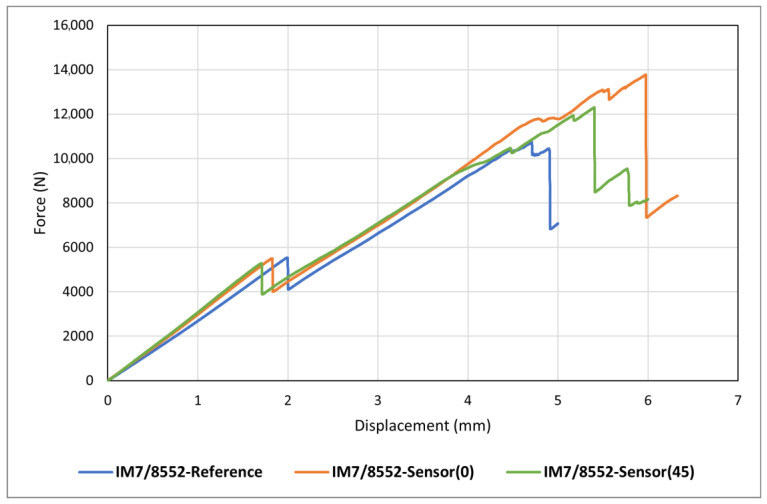
Static indentation results of all tested samples.

Figure 7 shows the results of *reference* and *sensor* samples tested under static indentation loading. It is seen that all curves follow the same general pattern (global behaviour), featuring a linear behaviour until a noticeable load drop, followed by a non-linear behaviour with a lower slop that reaches a second, more significant load drop, and then multiple smaller drops. This pattern has also been seen in static indentation testing of quasi-isotropic CFRPs by other researchers [16,19,21,31,32]. The graphs presented in this figure show the high repeatability and accuracy of both the test setup and the obtained results.

**Figure 8:**
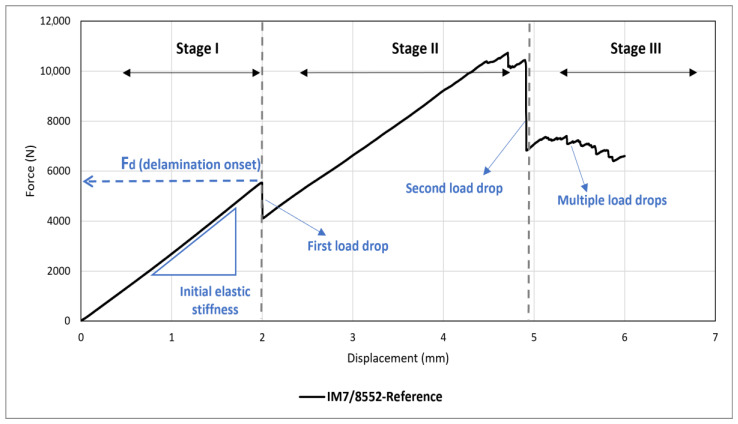
Different stages of damage initiation and propagation in a laminated quasi-isotropic CFRP composite under quasi-static indentation.

To further investigate global behaviour, the results of the first sample (IM7/8552-Reference) is chosen and shown in Figure 8. Three primary stages are evident from this figure. The first stage starts from the beginning of the test until a load drop occurs at displacement and force of approximately 2 mm and 6000 N, respectively. This initial phase represents the elastic region, characterised by a linear load–displacement response. The primary damage mechanism in this stage is matrix cracking, with the load drop marking the onset of delamination [21,33]. This delamination onset was distinctly identifiable during testing by an audible sound. The associated critical load at this point is shown by F_d,_ which is of great interest in this work. After the first load drop, a nearly linear response is seen until a second significant drop occurs at displacement and force of approximately 5 mm and 10,000 N, indicating more severe damage in the form of fibre failure. Alongside the load drop, this type of damage was identifiable by a distinct sound during testing. The minor load drops in the second stage, between the first and second significant load drops, are mainly associated with delamination propagation. Furthermore, the magnitude of the second load drop surpasses that of the first, indicating more severe damage to the specimen (see Table 2). Generally, damage in CFRPs is not visible until this point, and it only becomes visible on the surface after fibre failure happens. The third stage, initiated after the second load drop, signifies that the sample has incurred significant damage and cannot withstand further loading. Consequently, multiple small drops occur until the sample undergoes complete failure. Given that the objective of this research is to develop sensors capable of enhancing visual inspection of BVID, the emphasis will be on the first two stages. Accordingly, all subsequent low-velocity impact tests are designed to induce damage within these two regions.

#### 5.1.2. Influence of Adding Sensors on Indentation Properties

The sensor-to-substrate stiffness ratio is critical in designing bio-inspired mechanochromic hybrid composites [34]. Therefore, determining the extent to which the sensor affects the stiffness of the structure is crucial. Basic calculations can be conducted regarding the axial structural stiffness of both the sensor and substrate to assess the stiffness increase induced by the sensors. The most accurate results are typically achieved when employing low-structural stiffness sensors on high-structural stiffness substrates, as this minimises any substantial increase in substrate structural stiffness. In practice, the sensors must be made the thinnest and narrowest possible if the substrate has a relatively low structural stiffness [14]. In this case, the influence of adding sensors on the mechanical properties is experimentally studied here.

Figure 7 shows the load–displacement curves for all samples, including a reference sample (IM7/8552-Reference) and two sensor-integrated samples with different fibre orientation in the carbon sensing layer (IM7/8552-Sensor(0) and IM7/8552-Sensor(45)). Qualitatively speaking, adding a sensor on each side of the laminate appears to have marginally increased the structural stiffness of the samples. The structural stiffness alteration attributed to the sensor addition is negligible within the elastic region before the initial load drop, becoming more discernible beyond this point. A comparison between IM7/8552-Sensor(0) and IM7/8552-Sensor(45) samples suggests a slightly higher structural stiffness in the latter. This could be attributed to the alignment of the fibre orientation in the sensing layer and its adjacent carbon layer on the substrate, both positioned at 45°, thereby contributing to the sample’s increased structural stiffness. Another point highlighted by Figure 7 is that adding sensors can slightly change the first load drop and second load drop thresholds, meaning that the onset of delamination and final fibre failure is influenced by incorporating sensors. This is regardless of the sensor attachment method and fibre orientation of the sensing layer, as this trend can be observed in all graphs here, and in research by Fotouhi et al. [16], where the sensor and substrate were co-cured in a one-step process.

A summary of important information for a quantitative analysis that is obtained from indentation tests is presented in Table 2. The absorbed energy during an impact or indentation event at the first and second load drop can be attributed to the induced damage due to elastic and plastic deformations in the form of delamination and fibre failure, respectively. Therefore, comparing the absorbed energy of different samples can give interesting information about the damage mechanisms. The results indicate that the energy absorption at the first load drop across all samples is relatively consistent. However, there is notable variability in the maximum energy absorption values (observed at the second load drop). For instance, adding sensors could increase the maximum absorbed energy by up to 56% and 27% for sensors with sensing layer fibre orientation of 0° and 45°, respectively. The higher energy absorption in IM7/8552-Sensor(0) compared to IM7/8552-Sensor(45) suggests more significant induced damage, characterised by increased deformation and energy dissipation.

#### 5.1.3. Sensor Activation and Visual Inspection

**Figure 9:**
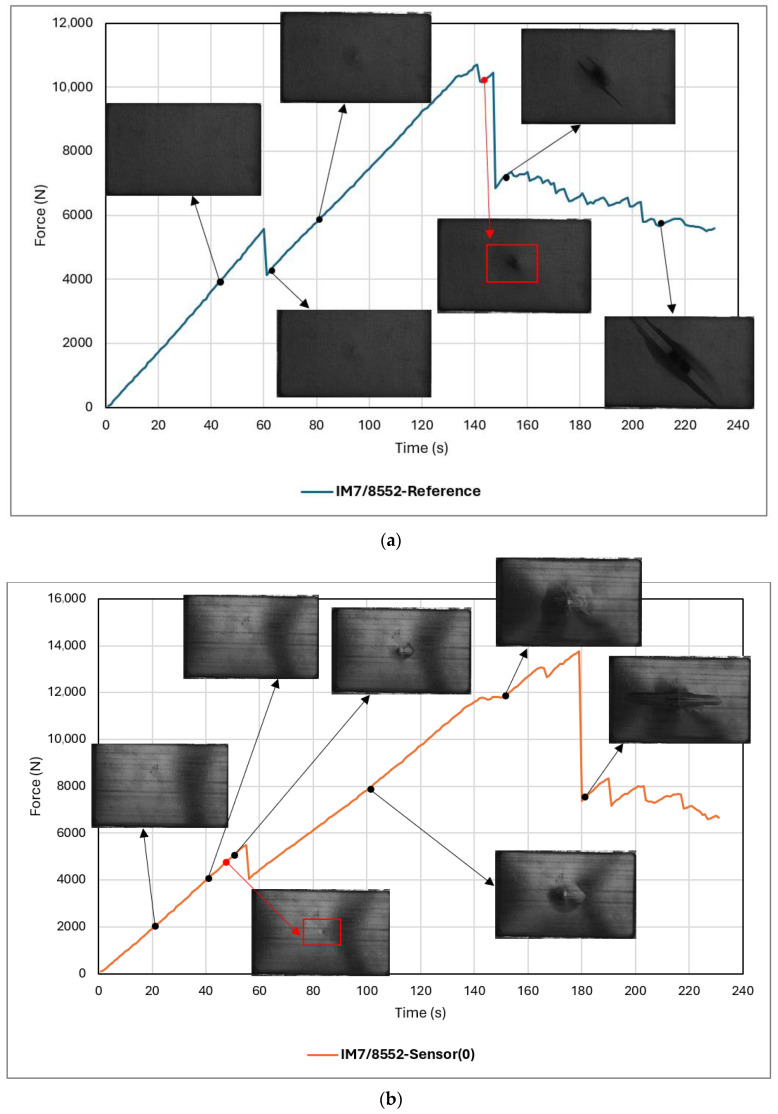

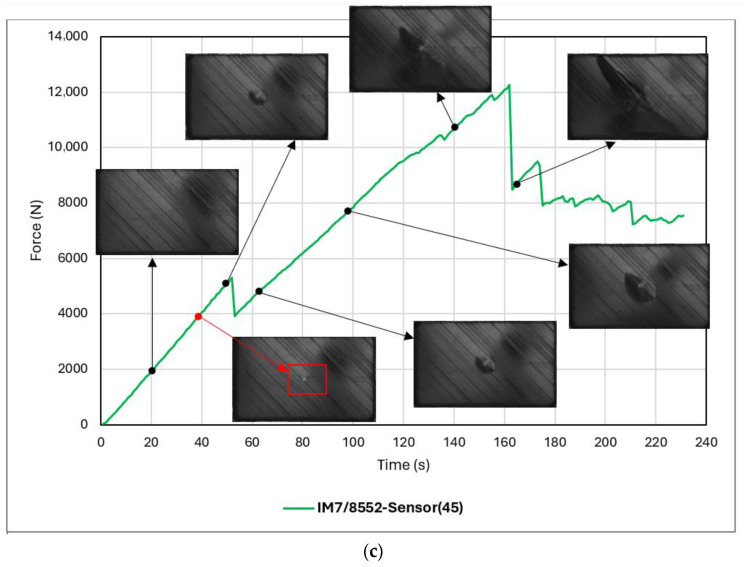
Visual inspection and sensor activation during static indentation experiment: (**a**) IM7/8552-Reference, (**b**) IM7/8552-Sensor(0), (**c**) IM7/8552-Sensor(45).

This section presents the visual inspection results, focusing on sensor activation and damage detection performance. For the scale, images of the back face cover the whole sample size (100 mm ×150 mm). As shown in Figure 9, damage on the back face remains imperceptible until just before fibre failure occurs at the second load drop, around forces of approximately 10,000 N. The initial point of visible damage is denoted by red colouring on the graph. This is where the structure has undergone significant damage and poses a very low residual structural integrity. However, across both two graphs associated with *sensor* samples, it is evident that the sensor on the back face activates before the first load drop at forces below 5000 N (highlighted by red colouring). This observation highlights the sensor’s ability to detect damages as subtle as matrix cracks, given that its activation precedes the initial load drop, thereby rendering it a viable early warning tool for BVID detection and avoiding delamination.

In Figure 9b,c the sensor on the back face activates before the first load drop, with the activated area expanding as the force increases. Upon analysing images captured during the indentation test of *sensor* samples, it was observed that the visible damage pattern on the surface enlarges up to a certain point, that is, where delamination has already happened, but fibre failure is yet to occur. However, beyond this point, the activated area exhibits minimal change despite increasing force. This suggests that in terms of detecting damage “levels” (rather than damage “existence”), the sensor is well suited for identifying low-energy damage types such as matrix cracks and delamination. This is evidenced by the lack of significant alterations in the appearance of the samples (size and shape of damage pattern) beyond a certain force threshold. Nonetheless, the sensor can also be utilised for “visualising” and “detecting” higher-energy damage types. Furthermore, a comparison between Figure 9b,c reveals sensor activation at a lower force for the sample with a sensing layer orientation of 45°. This suggests that the activation force threshold could be influenced by altering the fibre direction of the sensing layer.

When subjected to out-of-plane loading, the damage on the back face is tensile fibre failure because of the global bending effect, whereas the front face undergoes compressive fibre failure and local deformation due to local contact force. In thicker laminates, local damage tends to be predominant, whereas, in thinner laminates, such as those of this study, the global bending effect prevails, leading to more significant damage on the lower plies (back face). Consequently, initial failure in thin, flexible laminates typically occurs in the lower plies. Conversely, in thicker and stiffer laminates, initial failure occurs on the top surface due to contact stress [33,35]. This can explain an earlier sensor activation on the back face than the front face. This was also observed in other studies on the application of the hybrid sensor on CFRP and hybrid flax/carbon composites [36,37]. Note that the images showcased here were captured using a black-and-white camera available at the time of testing. For a more comprehensive interpretation of the images and to observe the colour changes induced by the sensor, refer to the post-indentation visual inspection images.

**Figure 10:**
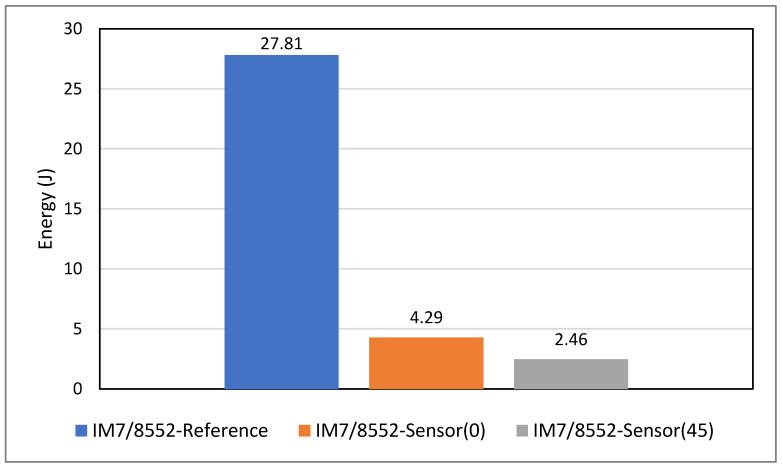
Threshold energies for visually detectable damage.

Figure 10 represents a comparison of threshold energies for detecting damage across all tested samples, highlighting the performance of the sensor in visualising damage on the back face at very low energies. It is observed that while damage on the back face of the IM7/8552-Reference sample is not visible until over 27J, it becomes visible in *sensor* samples at energies as low as less than 5J.

**Figure 11:**
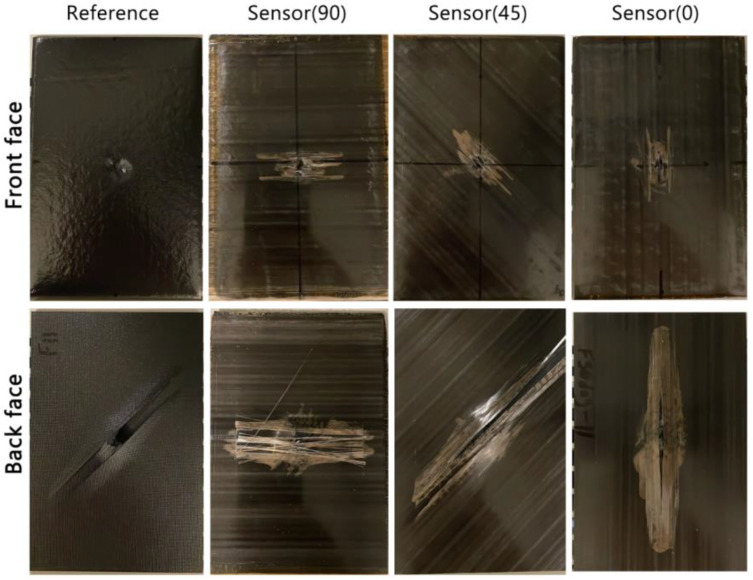
Post-experiment visual inspection.

The post-experiment inspection images are displayed in Figure 11. Note that in addition to the samples discussed previously, this figure includes visual inspection images of an IM7/8552-Sensor(90) sample. This test was conducted initially, and its force–displacement graph was not included in the preceding sections. Its purpose here is to illustrate a comparison of visual damage patterns at various degrees.

Various modes of composite failure are observed in these samples, stemming from indentation tests continuing until significant load drops are observed. Noticeable damage is evident on the front face of the *reference* sample. A recessed area is evident, where the indenter made contact with the specimen. Additionally, a series of small, stepped lines indicate cracks propagating in the upper carbon layer. On the back face, there is significant evidence of damage, particularly fibre failure at the centre of the image directly under the indenter, running parallel to the fibre direction. On the front face of *sensor* samples, light-coloured patches parallel to the sensor fibre direction indicate sensor activation. On the back faces, the significant light patch directly under the contact area of the indenter demonstrates good sensor activation. Above and below the activated patches, the sensor activation appears striped; this indicates the strain due to the tension on the back face has exceeded the strain to failure of the carbon sensing layer. This result was seen when sensor-integrated specimens were tested in tension by Czél and Wisnom [13]. Overall, the visual damage pattern is larger on the back face than the front face. The findings here suggest that incorporating sensors can lead to notable improvements in visual inspection by revealing visible damage patterns on both the top and back surfaces. Additionally, it enhances the load-bearing capacity, offering flexibility in design to meet the specific requirements of each application. This adaptability can be achieved by adjusting sensor-related parameters, such as the fibre orientation of the sensing layer, to suit desired outcomes.

### 5.2. Low-Velocity Impact

#### 5.2.1. Definition of BVID

**Figure 12:**
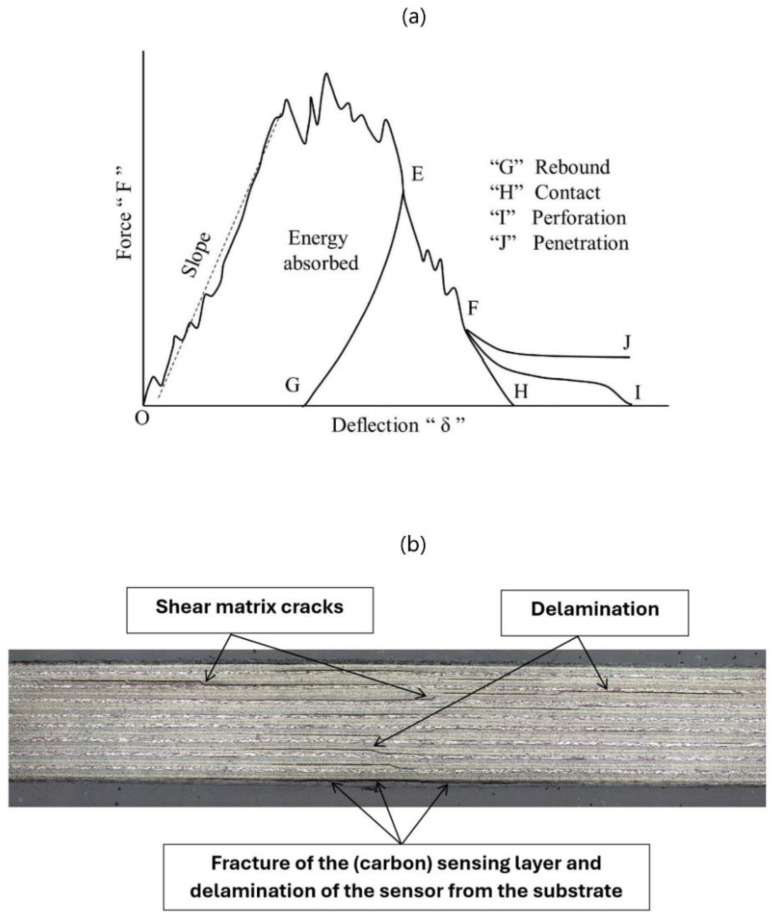
(**a**) Different damage scenarios with respect to permanent deflection (indentation) [38]. (**b**) The microscopy image depicts impact-induced damage at 12J, captured from the cross-section of the *sensor* specimen, demonstrating internal damage mechanisms, such as shear matrix cracks and delamination. Additionally, the fracture of the carbon sensing layer and delamination of the sensor from the substrate are evident, both at the point of impact on the front and back faces.

**Figure 13:**
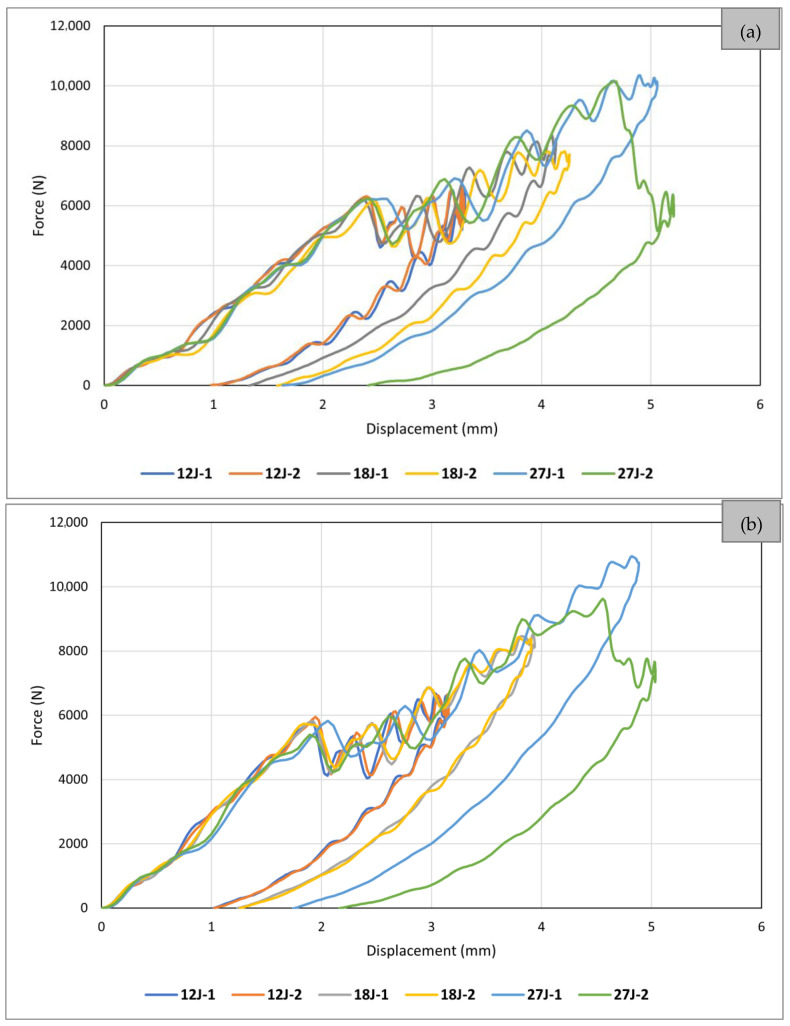
Low-velocity impact test results: (**a**) *reference*; (**b**) *sensor*.

Shah et al. [38] reviewed impact resistance and damage tolerance of FRP composites, summarising different damage scenarios with respect to permanent deflection (indentation) of the composite, including permanent indentation with rebounding of the impactor without perforation, with perforation and the penetration of the impactor (see Figure 12a). BVID can be defined using various metrics or standards. In accordance with general guidelines, permanent indentations ranging from 0.3 mm to 0.5 mm suggest the presence of BVID, while indentations of 2 mm or perforations of 20 mm indicate minor visible impact damage. As indicated in [16], the impact energy required to induce BVID was estimated to be 40% higher than the critical energy level derived from the indentation test, owing to strain-rate sensitivity. Nevertheless, other studies have presented diverse correlations between the critical energy level in quasi-static indentation and low-velocity impact tests [27,28,29,33,39,40,41,42,43,44,45]. This will be studied in more detail in Section 5.3 of this study. Hence, to guarantee the force is sufficient to induce delamination, 12J was considered as the minimum impact energy in this chapter. Additionally, for investigating impact behaviour at higher energy levels, 18J and 27J, were considered as the second and third impact energies for examination. Indentation results showed that for the IM7/8552-Reference sample, the energy required to induce fibre failure slightly exceeds 27J (see Table 2). Hence, this value was selected as the upper limit to study in this research. At these energies, samples undergo delamination; nonetheless, there is no perforation or penetration. This is indicated by a similar trend observed in the graphs obtained from the tests (see Figure 13) to the situation “G” depicted in Figure 12a. Furthermore, microscopy images of the cross-section of impacted samples confirm delamination occurring at the minimum considered impact energy (12J) (see Figure 12b). For the scale, the image shows the central 5 cm throughout the width. Nevertheless, surface damage is either invisible or barely visible on both impacted and non-impacted surfaces when viewed from a distance of 40 cm at a viewing angle of 0°, thus classifying the damage as BVID. Moreover, the measured dent depth data from the Alicona 3D profilometer averaged around 0.5 mm, showing minimal deviation across different groups. This confirms that for all samples, the dent depth aligns with the specified BVID range outlined in the aerospace sector [9]. Every individual impact coupon test underwent two repetitions to verify repeatability.

#### 5.2.2. Global Behaviour

The results of all impact tests are shown in Figure 13. It indicates that at impact energies of 12J and 18J, both *reference* and *sensor* samples exhibit excellent repeatability, as evidenced by the close alignment of the graphs. However, some discrepancies arise at the impact energy of 27J, particularly noticeable when approaching the maximum force and displacement. Notably, this trend persists in both *reference* and *sensor* samples at the 27J energy level. This can be attributed to two potential factors. Firstly, the influence of boundary conditions may become more pronounced as the impact energy increases. This is because, in the impact tests conducted in this investigation, variations in impact energy were achieved by altering the height from which the sample is dropped while maintaining a consistent weight. Consequently, at higher impact energies (27J), the sample undergoes a substantial increase in drop distance, intensifying the impact of boundary conditions on the dynamic response. This effect is notably more pronounced compared to lower energies (12J), where the distance between the impactor and the sample is minimal. Another factor contributing to the varying impact response at 27J energy could be related to the results obtained from the indentation tests. The energy required to induce fibre failure in the IM7/8552-Reference sample was measured at 27.81J, indicating that 27J serves as the threshold for failure occurrence. Therefore, upon examining the trends in the graphs in Figure 13, it can be inferred that at 27J, one instance denotes the sample having undergone fibre failure, as evidenced by a distinct change in its curve pattern, particularly after reaching maximum force (see 27J-2 graphs in both Figure 13a,b). However, in another instance, the sample sustained damages leading up to failure but did not reach failure, resulting in similar curve and drop patterns to lower energies (12J and 18J) across the entire displacement range (see 27J-1 graphs in both Figure 13a,b). This is confirmed by visual inspection results that will be presented in the next section. In line with the research objective, which focuses on developing sensors for BVID, throughout the remainder of this paper, the 27J-1 sample (the one without fibre failure) will represent the 27J energy in all graphs and tables, unless otherwise specified.

**Figure 14:**
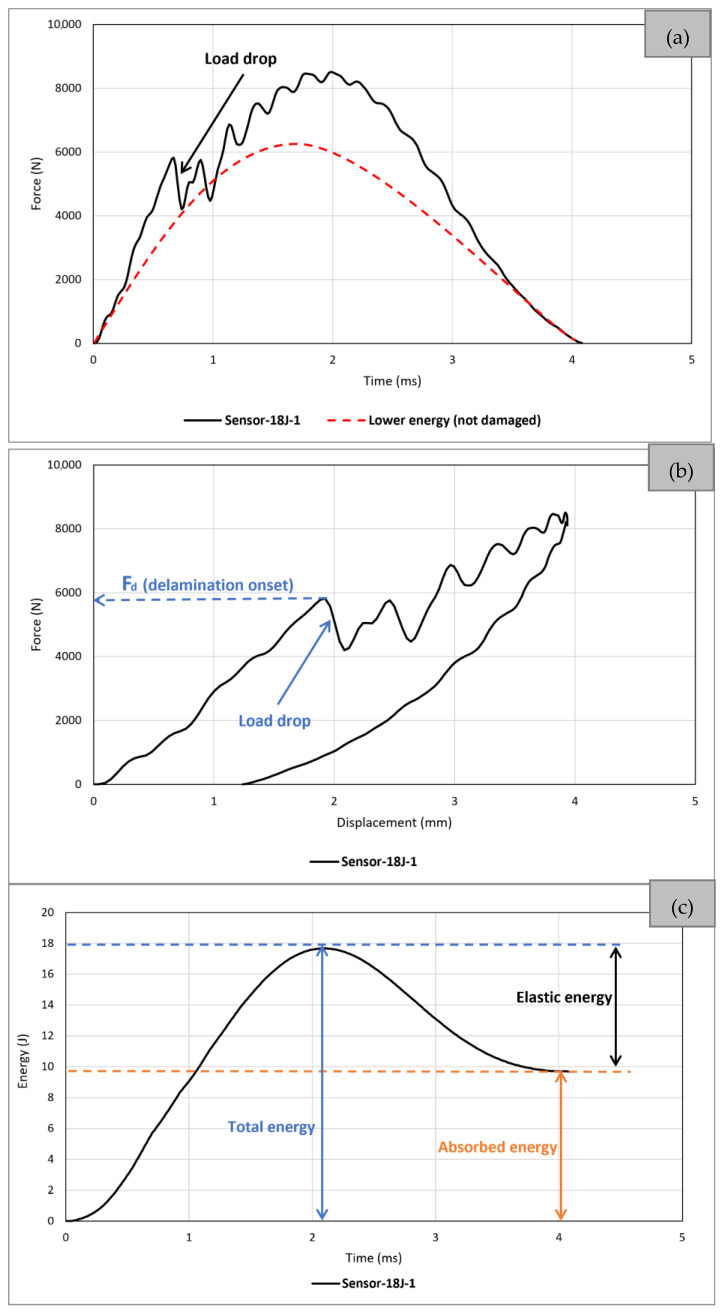
Typical low-velocity impact test results: (**a**) force–time, (**b**) force–displacement, (**c**) energy–time.

To comprehensively analyse the global behaviour of a CFRP under the impact, the force–time, force–displacement, and absorbed energy–time graphs of the *sensor* sample (18J-1) are depicted in Figure 14. In Figure 14a, the force–time results, directly recorded and obtained from the data acquisition system during the impact test, are illustrated. The graph can be divided into two distinct phases. The first half commences with the initial contact between the impactor and the specimen and extends until the velocity of the impactor reaches zero, indicating the point at which the impactor is about to rebound. Notably, a significant load drop associated with delamination is observable during this phase. A red graph corresponding to a hypothetical lower impact energy that does not cause any damage is also included, demonstrating no significant drop in load. The second half portrays the rebound process, concluding at the moment when the impactor completely separates from the sample. As damage is primarily incurred during the first half, multiple drops are evident in the graph, while the second half displays a smoother and less fluctuating trend. Figure 14b illustrates the force–displacement history, calculated from the force–time results. The graph exhibits an initial almost linear response from 0 to nearly 6000 N. Minor load drops in this region may be attributed to matrix cracking. Then, a sudden drop in load is observed, signifying the onset of delamination. Subsequently, intermittent minor drops occur until reaching a maximum force of slightly over 8000 N. Following this, once the impactor has fully transferred its energy to the specimen, there occurs a redirection of elastic energy stored within the specimen back to the impactor, inducing a rebound phenomenon. This transition is graphically represented by a gradual reduction in both force and deformation. The plot of the unloading curve passes under the loading section of the plot; this encloses an area that corresponds to the total absorbed impact energy. This energy is absorbed through both the dynamic response of the specimen and through damage mechanisms. The change in gradient and the enclosed area indicate that damage has occurred for this impact energy. Nevertheless, the enclosed nature of the graph means that there is no penetration or structural failure [38], which supports findings from both indentation and visual inspection. Figure 14c shows the energy–time history that can be divided into two parts: (a) elastic energy and (b) absorbed energy. The curve exhibits a progressive increase during loading, reaches a peak, decreases during unloading, and ultimately levels off at a constant value. The latter signifies the total energy permanently absorbed through damage mechanisms by the composite specimen at the end of the impact event. The peak of the curve corresponds to the respective impact energy (18J here).

#### 5.2.3. Influence of Adding Sensors on Impact Properties

**Figure 15:**
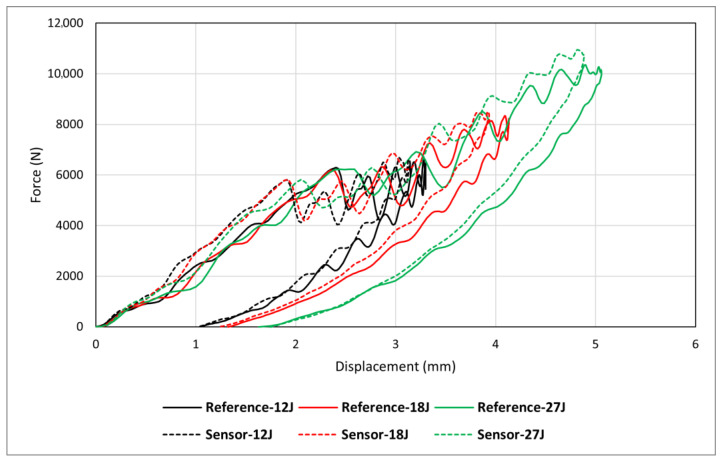
Influence of adding sensors at different impact energies.

**Figure 16:**
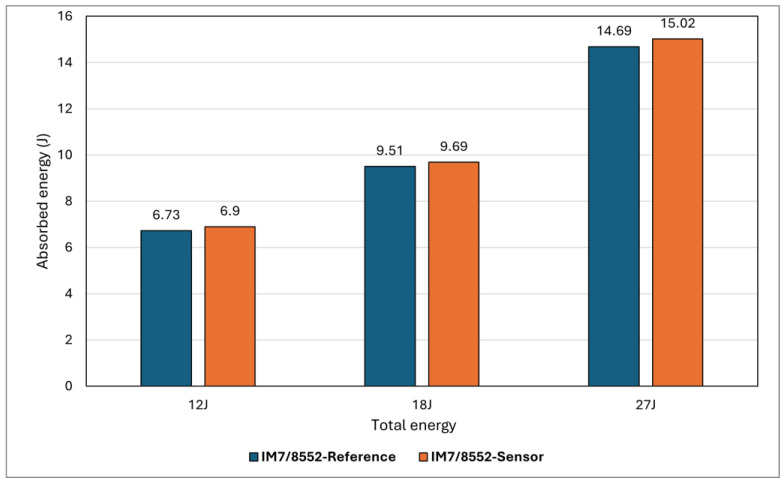
Comparison of the absorbed energy in all samples at various impact energies.

The assessment of composite materials’ impact resistance typically involves examining aspects such as impact energy absorption, resistance to damage (specifically, the extent of damage following a non-penetrating impact), and tolerance to damage (residual properties after non-penetrating impact) [46]. This research focuses on energy absorption and resistance to damage. Accordingly, it is essential to understand the influence of sensor incorporation on the mechanical properties of the substrate structure and minimise this effect to achieve a reliable self-sensing system. Figure 15 compares two groups of samples, *reference* and *sensor*, at three different impact energies, and important information obtained from the graph is presented in Table 3. The results demonstrate a higher maximum force at a relatively lower maximum displacement for *sensor* samples compared to *reference* ones, meaning that adding sensors slightly increased the rigidity and structural stiffness. Considering the maximum force, for example, the results suggest that adding sensors could increase the maximum force by approximately 1% at 12J energy, 2% at 18J energy, and 6% at 27J energy, indicating that the effect of adding sensors on impact behaviour appears to be more significant at higher impact energies. Overall, no significant difference can be seen between the two types of specimens, indicating the addition of sensors has little influence on the maximum force generated by the specimen, though it does slightly increase the energy absorption, suggesting the sensor may slightly enhance impact resistance in terms of the energy absorption capacity (see Figure 16). The post-impact visual inspection revealed that these drop energies had activated the sensor, meaning that energy was absorbed by damage within the sensing layer, which could be a possible reason for the increased energy absorption in *sensor* specimens. Overall, results suggest that within the BVID range, the sensor does not change mechanical properties significantly while improving the load-bearing capabilities (energy absorption) of the entire structure. This aligns well with the indentation test results.

#### 5.2.4. Ultrasonic C-Scan and Visual Inspection

**Figure 17:**
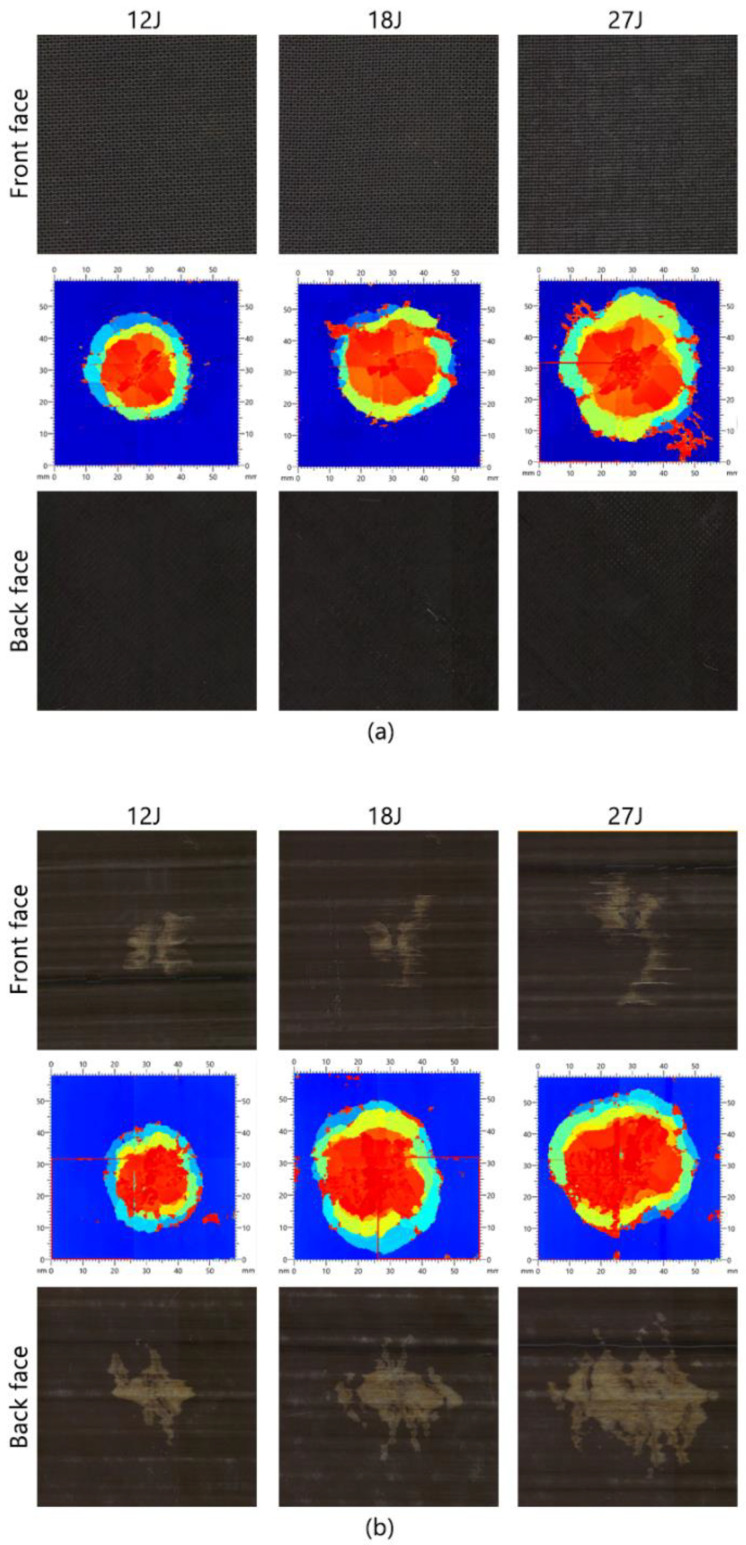
Ultrasonic C-scan and visual inspection results: (**a**) *reference*; (**b**) *sensor*.

No visible signs of impact damage on the back face are apparent in any of the three energies investigated in the *reference* samples. However, upon closer scrutiny of the 27J sample, a subtle indentation is discernible in the region directly affected by the impactor on the front face, suggesting internal damage within the specimen, indicative of BVID. This becomes more apparent upon physical examination. Ultrasonic examination, on the other hand, reveals evidence of internal delamination damage across all three energy levels, with the extent of damage increasing proportionally with energy input (see Figure 17a). In contrast to the *reference* samples, the visual inspection results of the *sensor* samples clearly reveal damage on both the top and back faces. This indicates that the sensor was triggered at all three energy levels, with the visual damage proportionately increasing as energy levels rose. Notably, the visual damage pattern on the back face differs from that on the front face due to different damage modes. On the back face, a discernible light patch suggests sensor activation due to tensile strain from the impact loading. Within this region, a darker speckled area indicates fibre fracture within the sensing layer, while a larger, lighter area signifies delamination of the glass layer from the carbon sensing layer. The front face displays less pronounced sensor activation, though it still exhibits a discernible activated area. With increased energy, the activated area expands more prominently in a direction perpendicular to the fibre direction. Additionally, short white lines surrounding the impacted area, aligned parallel to the sensor fibre direction, indicate matrix damage within the sensing layer. This is particularly noticeable in the case of 27J energy (see Figure 17b). For the scale, all images in this figure cover a dimension of 50 mm × 50 mm.

**Figure 18:**
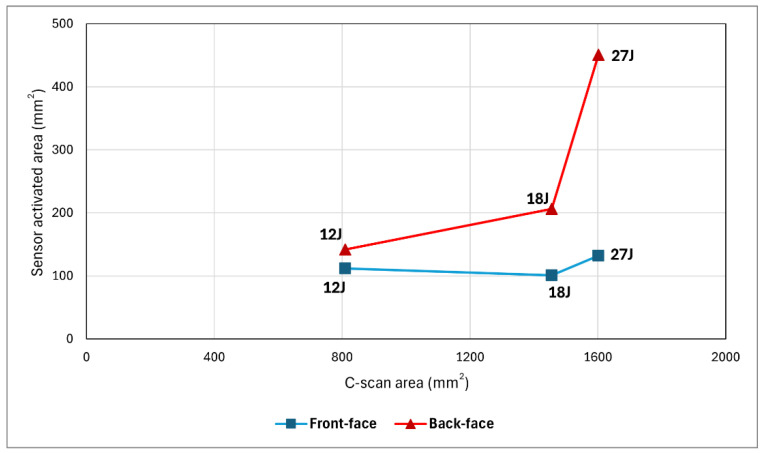
Comparison of the surface visible damage area and C-scan damage area at three impact energies.

Figure 18 compares the size of the visual damage area on the surface and the size of the C-scanned damage area for the *sensor* samples at the three impact energies. It is evident that as the impact energy increases, both the C-scanned and sensor-activated areas on the surface also increase. However, there is an exception with the activated area on the front face of the 18J sample. Note that the values on the vertical axis of this graph represent the activated area size, which is smaller for the front face at 18J compared to 12J due to its inconsistent elongated length but relatively shorter width, unlike the 12J sample, which covers a consistently smaller area. Nevertheless, the visible damage in the 18J sample is still more significant than in the 12J sample. Overall, this graph suggests that the activated damaged area on the surface can serve as an estimate of the severity of internal damage.

### 5.3. Comparison of Low-Velocity Impact and Quasi-Static Indentation

**Figure 19:**
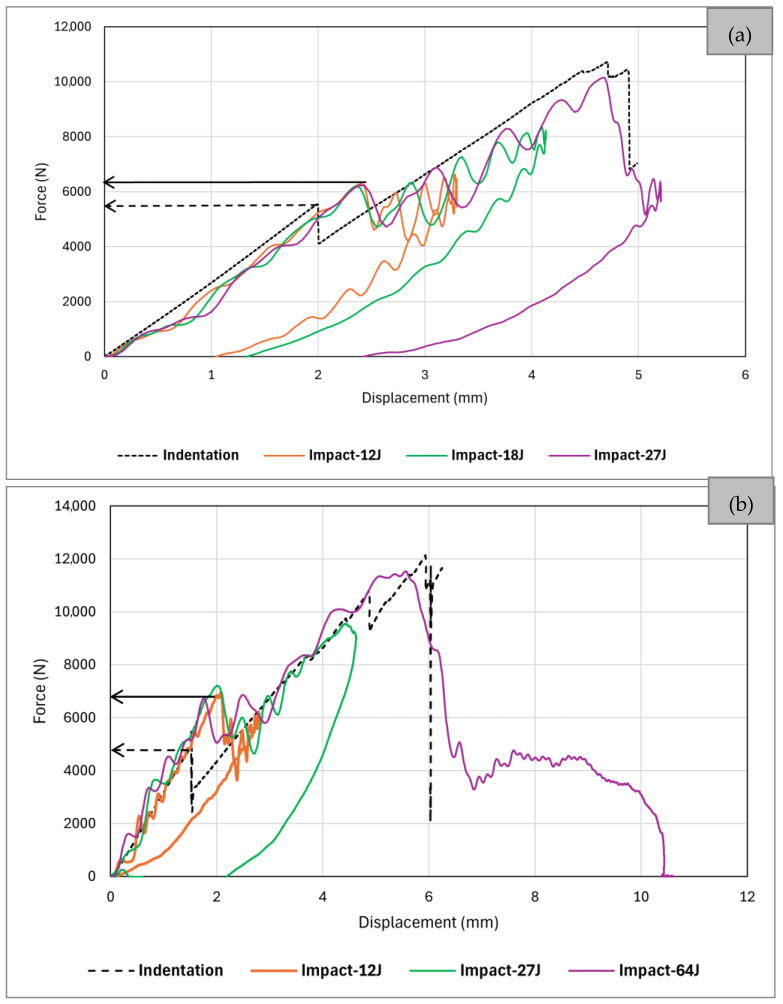
Comparison of indentation and impact response: (**a**) IM7/8552 samples of this research (*reference* samples); (**b**) T800/MTM49-3 samples of work by Fotouhi et al. [16].

Given the dynamic nature and short duration of low-velocity impact tests, analysing the progression and development of degradation mechanisms within a structure during such tests proves challenging and can mainly occur post-test completion. Consequently, researchers have redirected their attention to the static indentation test, which has demonstrated similar overall behaviour and resulting damage states compared to low-velocity impact tests [27,28,29,33,39,40,41,42,43,44,45]. The static indentation test offers the advantage of pausing the test at different stages, allowing for the observation of damage evolution. Nonetheless, it is crucial to thoroughly examine the similarities and disparities between these two tests to gain a deeper understanding of their correlation and the potential for substituting a low-velocity impact test with a quasi-static indentation test. Figure 19a compares force-displacement curves for the indentation and impact tests of this research. Note that the curve labelled “27J” in Figure 19a corresponds to the “27J-2” curve depicted in Figure 13a. This choice was made because the “27J-2” sample experienced fibre failure, enabling a comparison of fibre failure in both indentation and impact tests. Different parameters can be considered to compare the quasi-static indentation and low-velocity impact tests, such as the dent depth, the initial stiffness (before the first load drop), the percentage of the first load drop, critical delamination load (at first load drop), the delamination size, and the fibre failure load (or its equivalent absorbed energy). Abi Abdellah et al. [41] reported that during low-velocity impact tests, the total energy exceeded that of static tests for the same impactor displacement. However, the absorbed energy remained consistent between static and dynamic scenarios, indicating an equivalence in terms of damage under comparable displacement conditions. This finding was supported by observations made through X-ray analysis, microscopic examination, and the evolution of crack lengths. Sun and Hallett [22] conducted both tests on quasi-isotropic composites with ply-block scaling and sub-laminate scaling configurations (the latter is similar to specimens used in this investigation). Some indentation tests were paused at different stages of damage progression, and X-ray CT scanning and ultrasonic C-scan tests were conducted to compare the delamination patterns with matrix cracks in neighbouring plies in both tests. The overall damage morphologies showed remarkable similarities regarding individual delamination shapes, the number of major matrix cracks, interactions between matrix cracks and delamination, bottom transverse crack length, and delamination-free zone size. Given the high similarities between the two loading scenarios at both interlaminar and ply levels, they inferred that the interaction mechanism between matrix cracks and delamination is governed by the plate response under transverse loading, regardless of the loading rate employed during testing. The permanent indentation depth resulting from the quasi-static indentation test was larger than that from the impact test for equivalent energy absorption levels. Furthermore, it was noted that, for a specified delamination area, the indentation depth is greater in indentation tests than in impact tests. This could be attributed to the significantly longer duration of transverse loading in static tests compared to impact tests, resulting in less energy dissipation through other mechanisms. Similar results about the comparison of the permanent indentation between the two tests were reported in [42].

Herein, a comparison of the critical delamination load, the initial stiffness, and fibre failure load obtained from the test results of this investigation are provided (see Figure 19a). This comparison is also extended to the test results reported by Fotouhi et al. [16], as depicted in Figure 19b. The inconsistency in critical delamination loads between indentation and impact tests is readily apparent, with the critical loads in static indentation tests consistently lower than those in impact tests. Additionally, the critical load for impact tests under various energy levels appears to align closely, all approximately 6300 N, whereas the equivalent static load is nearly 5500 N. The critical indentation and impact loads shown by Figure 19b are 4900 N and 6900 N, respectively. These align with findings reported in [22,39,41]. Sun and Hallett [22] also observed a similar trend, reporting an average increase of nearly 30% in the ply-block scaling samples and 40% in the sub-laminate scaling samples for dynamic critical load compared to static critical load. Hence, it can be concluded that the increase in critical load under dynamic loading is independent of impact energy, sub-laminate scaling (ply-block), and laminate overall thickness for a given material system. Moreover, there appears to be variation in the percentage of load drop. However, due to significant vibrations and the dynamic effects of the plate during the loss of stiffness, the level of load drop in impact cases directly taken from force plots may not be entirely accurate.

Both Figure 3 and Figure 19a,b demonstrate a high level of similarity in the initial stiffness of the load–displacement curves obtained from the static and dynamic tests. This observation indicates that both displacement and force measurements obtained from static indentation and impact tests are precise. Therefore, the higher dynamic critical load observed in impact tests may not be due to misinterpretation or measurement errors. The disparity is believed to reflect the influence of loading rate on alterations in material intrinsic properties. Consequently, the increase in the critical load in the impact test is more likely a consequence of the strain rate dependence of the mode II critical strain energy release rate.

The critical load corresponding to fibre failure (second significant drop) in the indentation test closely mirrors that of impact tests in Figure 19a,b. In Figure 19a, this load drop for both indentation and impact tests occurs at a force and displacement of approximately 10,000 N and 5 mm, respectively, while in Figure 19b, these values are 12,000 N and 6 mm, respectively. The calculated absorbed energy values from the area under the force–displacement curve of the indentation tests for both figures were slightly higher than 27J. Thus, an impact energy of 27J or higher was anticipated to result in fibre failure. Notably, the impact energy of 27J in Figure 19a has caused this damage, indicated by a recognisable drop, while in Figure 19b, the impact energy of 64J has led to fibre failure and perforation, evidenced by a force–displacement graph similar to scenario “I” in Figure 12a. When examining the trends of second load drops, it is apparent that the values associated with these drops consistently align between the indentation and impact tests. This observation suggests that forces linked to fibre failure also appears to exhibit similarities between the two tests. Overall, the comparative study presented in this section, along with the findings from the literature, confirm that static indentation properly represents the barely visible damage induced in impact tests of quasi-isotropic laminates, offering a controlled, reliable, and repeatable loading setup, as well as stable damage growth [22,29,39].

## 6. Conclusions

This paper presented a design, manufacture, and application of hybrid thin-ply glass/carbon sensors to detect BVID in composite substrate structures. The design and performance of the sensor were further investigated using sensors with different fibre orientations. First, quasi-static indentation tests were conducted. Given its static nature, this test allowed for monitoring damage initiation and propagation and sensor activation during the test. This was achieved by taking images every second using a camera. Moreover, indentation tests provided insight into the minimum required energy to induce delamination damage, which was used to determine impact energies for the low-velocity impact tests. After completing impact tests, destructive and non-destructive evaluation methods were applied to analyse the internal damage and compare it with the damage on the surface. The non-destructive methods included visual inspection and ultrasonic C-scan, and the destructive method was to cut the impacted samples and analyse the microscopy images of the cross-section to evaluate the internal damage.

- Both indentation and impact test results showed that adding sensors on the two sides of a composite sample slightly changes its mechanical properties. A slight increase in absorbed energy was observed in the *sensor* samples compared to the *reference* samples. The changes in the fibre direction of the sensing layer affects the out-of-plane loading response, indicating that the effect of adding sensors on mechanical properties can be managed by adjusting this parameter through an appropriate design.
- Visual inspection results demonstrated the effectiveness of the sensor in visualising damage under both static indentation and impact loadings. Regarding the indentation test, the sensor on the back face activates before the first load drop, which is considerably earlier than the activation on the front face. This can stem from different damage mechanisms on each side. Moreover, applying the sensor could decrease the threshold energy for visually detectable damage on the back face from nearly 27J in a *reference* sample to less than 5J in *sensor* samples. Furthermore, changing the fibre orientation of the sensing layer influences the sensor activation threshold load, though it does not influence the shape or size of the visual damage pattern.
- C-scan and microscopy images confirmed that impacted samples at all studied energies had undergone delamination damage. Nevertheless, in *reference* samples, the damage was barely visible on the front face of 27J samples and not visible on the front face and back face of all other samples. In *sensor* samples, however, the impact-induced damage could be detected at energies as low as 12J, with the size of the activated area increasing in-line with the impact energy. Different sensor-activated patterns and sizes on the back face and front face of the *sensor* samples signified different damage modes on each side, with the back face showing a larger activated area at all energies.
- Comparing the C-scan and visual inspection images at different impact energies provided a correlation between the size of the internal damage and surface visible damage.
- Finally, a comparison was conducted between the results of the indentation and impact tests to evaluate their degree of similarity as potential substitutes. It was observed that despite a lower delamination threshold energy in the indentation test compared with the impact test, the static indentation effectively captures the barely visible damage induced in impact tests of quasi-isotropic CFRP laminates.
- Building on the foundation of sensor design and application established here, further studies could expand into other areas. For instance, the sensors might be applied to structures that undergo regular thermal cycling [47], such as hydrogen storage tanks or wind turbine blades. Additionally, the sensors may face extreme temperatures in certain applications, potentially compromising their functionality and integrity. Future research can, therefore, examine how real-world environmental conditions affect the sensors and their sensing performance. Another practical issue to address is the effect of multiple impacts, a scenario commonly encountered in real-world applications and extensively discussed in the literature [48]. Investigating how the sensors perform under repeated impacts and exploring design improvements, such as integrating self-healing capabilities with microcapsules, could enhance their durability and functionality.

## Figures and Tables

**Table 1:** Cured ply properties of the prepregs [16,19,26].

Prepreg	Cured Ply Thickness (mm)	Strain to Failure (%)	Tensile Modulus (GPa)
**IM7 carbon/8552 epoxy** [19]	0.125	1.6	161
**S glass/913 epoxy** [26]	0.153	3.7	45.6
**YS-90A carbon/epoxy** [16]	0.070	0.5	520

**Table 2:** Summary of important information obtained from indentation tests.

Sample	First Load Drop (Delamination)				Second Load Drop (Fibre Failure)			
	Force (N)	Displacement (mm)	Energy Absorbed (J)	Load Drop Rate (%)	Force (N)	Displacement (mm)	Energy Absorbed (J)	Load Drop Rate (%)
**IM7/8552-Reference**	5542.28	1.99	5.43	25.85	10445.30	4.89	27.81	34.63
**IM7/8552-Sensor(0)**	5506.07	1.824	5.01	27.12	13780.18	5.97	43.37	46.73
**IM7/8552-Sensor(45)**	5284.29	1.70	4.498	26.47	12304.06	5.40	35.24	31.05

**Table 3:** Summary of important information obtained from impact tests.

	Maximum Force (N)	Maximum Displacement (mm)	Total Energy (J)	Elastic Energy (J)	Absorbed Energy (J)
**Reference-12J**	6626.23	3.29	12	5.27	6.73
**Reference-18J**	8340.01	4.13	18	8.49	9.51
**Reference-27J**	10,347.64	5.05	27	12.31	14.69
**Sensor-12J**	6686.51	3.14	12	5.10	6.90
**Sensor-18J**	8509.39	3.93	18	8.31	9.69
**Sensor-27J**	10,949.49	4.88	27	11.98	15.02

## Data Availability

The data is available upon request.
